# Histone Methylation Inhibitor DZNep Ameliorated the Renal Ischemia-Reperfusion Injury *via* Inhibiting TIM-1 Mediated T Cell Activation

**DOI:** 10.3389/fmed.2020.00305

**Published:** 2020-07-10

**Authors:** Jiawei Li, Yue Qiu, Long Li, Jiyan Wang, Yin Celeste Cheuk, Ruirui Sang, Yichen Jia, Jina Wang, Yi Zhang, Ruiming Rong

**Affiliations:** ^1^Department of Urology, Zhongshan Hospital, Fudan University, Shanghai, China; ^2^Shanghai Key Laboratory of Organ Transplantation, Shanghai, China; ^3^Department of Critical Care Medicine, Zhongshan Hospital, Fudan University, Shanghai, China; ^4^Department of Urology, Shanghai Ninth People's Hospital, Shanghai Jiaotong University School of Medicine, Shanghai, China; ^5^Biomedical Research Center, Institute for Clinical Sciences, Zhongshan Hospital, Fudan University, Shanghai, China

**Keywords:** renal IRI, DZNep, TIM-1, T cells, NF-κB

## Abstract

Renal ischemia-reperfusion injury (IRI) after renal transplantation often leads to the loss of kidney graft function. However, there is still a lack of efficient regimens to prevent or alleviate renal IRI. Our study focused on the renoprotective effect of 3-Deazaneplanocin A (DZNep), which is a histone methylation inhibitor. We found that DZNep significantly alleviated renal IRI by suppressing nuclear factor kappa-B (NF-κB), thus inhibiting the expression of inflammatory factors in renal tubular epithelial cells *in vivo* or *in vitro*. After treatment with DZNep, T cell activation was impaired in the spleen and kidney, which correlated with the downregulated expression of T-cell immunoglobulin mucin (TIM)-1 on T cells and TIM-4 in macrophages. In addition, pretreatment with DZNep was not sufficient to protect the kidney, while administration of DZNep from before to after surgery significantly ameliorated IRI. Our findings suggest that DZNep can be a novel strategy for preventing renal IRI following kidney transplantation.

## Introduction

Ischemia-reperfusion injury (IRI) is the leading cause of renal function impairment after renal transplantation. It can induce a series of pathological phenomena, including renal inflammation and fibrosis, and eventually lead to the loss of kidney graft function or acute or chronic rejection, which affects the patient's quality of life (1–3).

The adaptive immune system plays an essential role in renal IRI (4–8). T cells mediate IRI or rejection, impair the recovery of kidney grafts, and even induce renal interstitial fibrosis. Therefore, inhibiting the activation of T cells has the potential to promote the functional recovery of kidney grafts.

T-cell immunoglobulin mucin (TIM) molecules, or kidney injury molecules (KIM) when expressed in the kidney, are costimulatory molecules critical in regulating adaptive immunity. There are eight TIM genes in mice (TIM-1 to 8) and three in humans (TIM-1,3,4) encoded by the TIM family (9). Recent evidence (10) has indicated the high expression of TIM-1 on the surface of activated CD4^+^ T cells after liver IRI. When using a TIM-1 monoclonal antibody (RMT1-10) to block TIM-1 function, T cell activation was reduced significantly. In RAG^−/−^ mice (T cell deficient), RMT1-10 had no effect on liver IRI. A similar result was also found in mice with renal IRI (11). Such reports reveal that TIM-1 is highly correlated with T cell activation. Thus, TIM-1 could be a potential target for intervention in T cell-mediated injury.

Recently, research has focused on epigenetic modifications. Epigenetic modifications are critical for protecting the function of grafts (12). Histone methylation, a common epigenetic modification, takes part in cell proliferation and differentiation. It has been reported that the histone methyltransferase Ezh2 mediates the functional alteration of T cells (13) and drives them to differentiate into various subtypes and impair their activation and proliferation (14). Acting as an inhibitor of Ezh2, 3-Deazaneplanocin A (DZNep) has been used to treat leukemia (15, 16), tumors (17), and other malignant diseases. Recent research has broadened its potential therapeutic effects in ischemic brain injury by promoting microglial activation (18) and tubulointerstitial fibrosis by inhibiting the expression of fibrogenic genes. It has become an ideal medication for epigenetic therapy owing to its reversible effects on gene expression. A previous study found that DZNep had an inhibitory effect on graft-versus-host disease (GVHD) in a kidney or bone marrow transplantation model, and it can only induce apoptosis of activated T cells but has no effect on naïve T cells (19).

In this study, we demonstrated that DZNep treatment can alleviate renal IRI in mice by inhibiting T cell activation through direct and indirect pathways. The indirect pathway may involve the impairment of interactions between T cells and macrophages by the TIM-1–TIM-4 axis. DZNep may have a better protective effect when used postoperatively. Our findings suggest that DZNep can be a novel strategy for preventing renal IRI following kidney transplantation.

## Methods

### Mice and Model of Renal IRI

Male C57BL/6 mice (20**–**25 g) were obtained from Shanghai SLAC Laboratory Animal Co., Ltd. Renal IRI was performed as follows: briefly, the bilateral renal pedicles were occluded for 45 min using non-traumatic clamps, and mice were sacrificed 36 h after reperfusion. For survival rate analysis, mice were observed for 7 d, and euthanized. Before surgery, mice were randomly divided into six groups (*n* = 6) according to the different treatments: (1) sham group (without renal pedicle occlusion); (2) DZNep group (DZNep treated without renal pedicle occlusion); (3) IRI group (with renal pedicles occlusion); (4) IRI+DZNep pretreatment group (mice were pretreated with DZNep for 2 days before surgery); (5) IRI+DZNep post-treatment group (mice were pretreated with DZNep immediately after reperfusion and 1 d after surgery); (6) IRI+DZNep group (mice were treated with DZNep for 2 days before surgery, right after reperfusion, and 1 d after surgery). DZNep (100μL 1 mg/kg) was subcutaneously injected at the bilateral inguinal area every time. DZNep was purchased from the National Cancer Institute (NCI), dissolved in sterile phosphate-buffered saline, and stored at −20°C. The experimental protocols were approved by the Animal Ethical Committee of Zhongshan Hospital, Fudan University.

### Assessment of Renal Damage

Serum creatinine (SCr) and blood urea nitrogen (BUN) levels were measured with an autoanalyzer (HITACHI 7600 P automatic biochemical analyzer [Japan]). Renal specimens were fixed with a 10% buffered formalin solution and hematoxylin and eosin (H&E) stained to determine histological injury. Ten random sections per slide (200×) were evaluated. Renal damage was graded as 0–4 based on the percentage of damaged tubules of each sample as previously described (20). Injury included cell vacuolization, cell necrosis, and interstitial infiltration.

### Immunohistochemistry

Fixed kidney sections were dewaxed, rehydrated, and incubated with either myeloperoxidase (MPO) to assess neutrophil infiltration (1:200, Thermo Fisher) or CD4 monoclonal antibody for T cells (1:500, Abcam).

### Cell Culture

The renal tubular epithelial cell line (TCMK-1 cells) was obtained from the ATCC® CRL-3216™American Type Culture Collection (Manassas, VA).

Cells were cultured in Dulbecco's modified Eagle medium F12 (Gibco, NY, USA) supplemented with 10% fetal bovine serum (Gibco, NY), 100 IU/mL penicillin, and 100 μg/mL streptomycin. Mouse splenic cells were ground and filtered from mouse spleens, and naïve CD4^+^ T cells were separated by microbeads (Miltenyi Biotec, NY) and later cultured in RPMI 1640 medium (Gibco, NY). Cells were maintained in a humidified atmosphere containing 5% CO_2_ and 95% O_2_ at 37°C. Splenic cells were further stimulated with ConA and seeded in 96-well plates (1 × 10^5^ cells/well) in the absence or presence of DZNep for 3 days. The proportion of CD4^+^CD69^+^ T cells was evaluated by flow cytometry.

### Hypoxia/Reoxygenation (H/R) Model and Treatment

For H/R stimulation, TCMK-1 cells were cultured in glucose/serum-free medium under hypoxic conditions (94% N_2_, 1% O_2_, and 5% CO_2_) for 24 h, followed by 2 h in normal media and normoxic conditions (5% CO_2_ and 95% O_2_). Cells in the control group were incubated under normoxic conditions. Cells in the DZNep group were pretreated with DZNep (40 μM) for 24 h followed by H/R stimulation.

### Cell Isolation and Flow Cytometric Analysis

To obtain a splenic single cell suspension, the mouse spleen was ground and filtered. The kidneys were cut into small pieces and digested in Hank's Balanced Salt Solution (Gibco) supplemented with 10% type IV collagenase (Gibco) and 0.002% DNase I (Gibco) at 37°C for 30 min. Dissociated cells were filtered and centrifuged at 1,000 rpm. Splenic and renal kidney cells were suspended in PBS+10% FBS (Gibco) and incubated with CD4-PE (eBioscience, CA), CD69-APC, TIM1-FITC antibody (BioLegend, CA), TIM4- PerCP-eFluor 710, and F4/80-FITC (Thermo Fisher Scientific) at 4°C for 30 min. After washing, flow cytometric analyses were performed using a FACScan and Canto cytometer (BD Biosciences).

### Realtime PCR

Total RNA was extracted from kidney tissue using TRIzol (Invitrogen Life Technologies) and transcribed into cDNA using HiScript II Q RT SuperMix for qPCR (Vazyme). Realtime PCR was performed using the iQ5 Real-time PCR instrument (Bio-Rad) with a SYBR green PCR mix (Yeasen). Gene expression levels were normalized to the GAPDH gene. The primer sequences are listed as follows (mice, 5′ ‘-3’′): interleukin (IL)-6, F: CTGCAAGAGACTTCCATCCAG, R: AGTGGTATAGACAGGTCTGTTGG; IL-10, F: CTTACTGACTGGCATGAGGATCA, R: GCAGCTCTAGGAGCATGTGG; IFN-γ, F: GCCACGGCACAGTCATTGA, R: TGCTGATGGCCTGATTGTCTT; TNF-α, F: CAGGCGGTGCCTATGTCTC; R: CGATCACCCCGAAGTTCAGTAG; KIM-1, F: ACATATCGTGGAATCACAACGAC; R: ACTGCTCTTCTGATAGGTGACA; and neutrophil gelatinase-associated lipocalin (NGAL), F: TGGCCCTGAGTGTCATGTG, R: CTCTTGTAGCTCATAGATGGTGC.

### ELISA

Whole blood of mice was centrifuged for 4,000 rpm for 5 min to obtain serum. KIM-1 expression in serum was detected using a TIM-1 (HAVCR1) Mouse ELISA Kit (Thermo Fisher).

### Western Blot Analysis

Total protein was extracted using lysis buffer and a 1% protease inhibitor cocktail. After centrifuging for 15 min at 12,000 g, the supernatant was transferred to a new tube and stored at −80°C. A Bradford protein assay kit was used to determine the concentration of total protein. Total protein was separated on SDS-poly-acrylamide gels, transferred to polyvinylidene difluoride membranes, blocked with 5% non-fat dried milk, and incubated overnight with KIM-1 (eBioscience), anti-IKKα, anti-NF-κB, anti-p-NF-κB, p-IκB (Cell Signaling Technology, Beverly, MA, USA, 1:1000), and GAPDH (Abcam, Cambridge, UK,1:1000). The membranes were washed with TBST and probed with HRP goat anti-rabbit IgG (H+L) cross-adsorbed secondary antibody (Thermo Fisher Scientific, 1:10000). Proteins were visualized and captured using a chemiluminescence image analysis system (Tanon, 5200 Multi).

### Statistical Analysis

GraphPad Prism 10 was used for data analysis. Data are presented as the mean ± standard error of the mean (SEM). A two-tailed independent Student's *t*-test was conducted after a demonstration of the homogeneity of variance with an *F*-test. The survival endpoints were analyzed using the Kaplan-Meier method. A *P* < 0.05 was defined as statistically significant.

## Result

### DZNep Alleviated Renal Dysfunction After Ischemia-Reperfusion Injury

**Figure 1:**
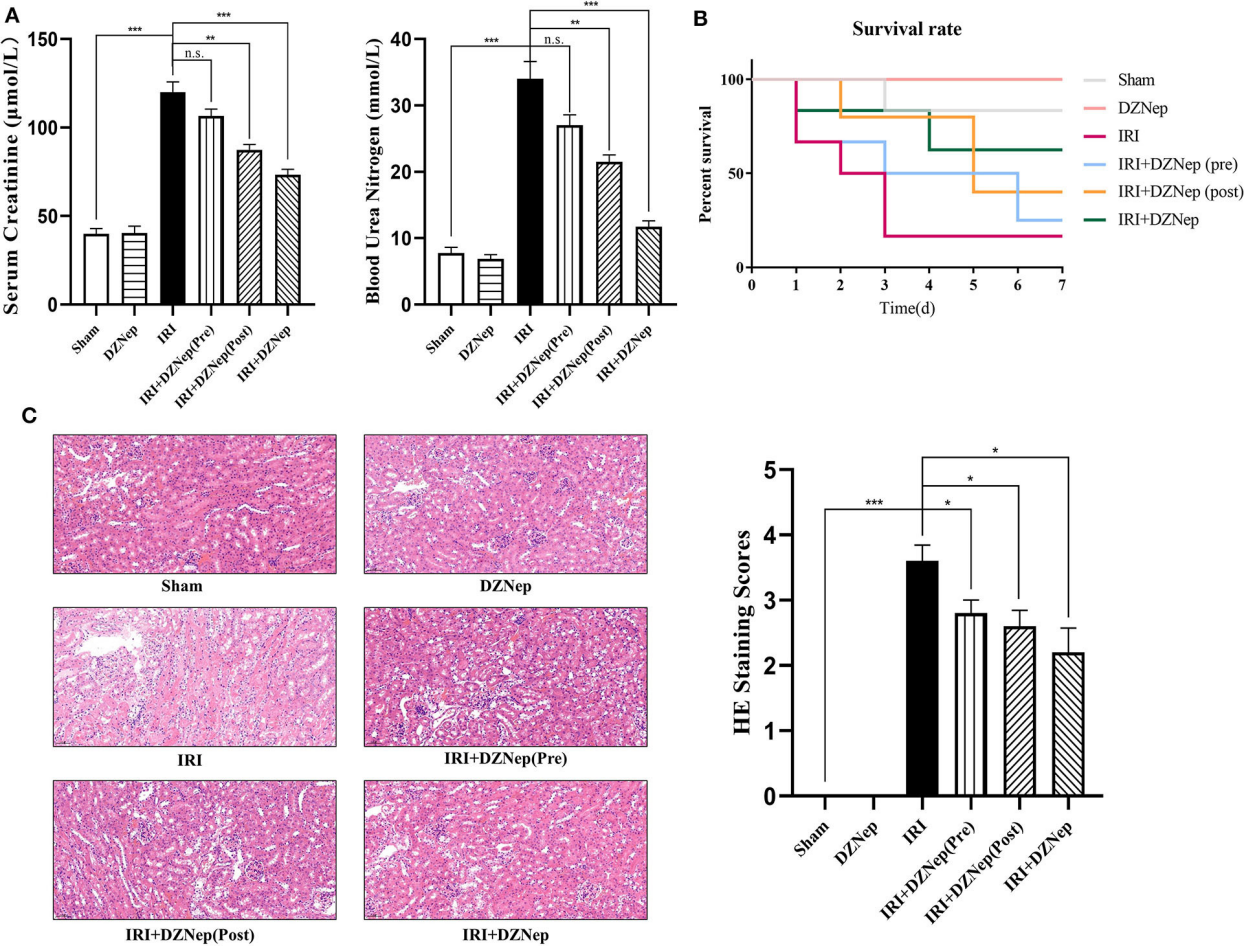
DZNep alleviated renal dysfunction after IRI. **(A)** Serum of mice in each group were tested for serum creatine (SCr) and blood urea nitrogen (BUN) level (*n* = 6). **(B)** 72-h survival rate in each group (*n* = 6). **(C)** Tubular injury level in different group indicated by H&E staining. Tubular injury scoring was made and shown at the right (*n* = 6). ^n.s.^*P* ≥ 0.05, *P < 0.05, **P < 0.01, ***P < 0.001.

We first evaluated the renal protective effect of DZNep in renal IRI and optimal administration time. DZNep was applied preoperatively, postoperatively, or from before to after surgery (details are shown in the Methods section). The SCr and BUN concentrations were measured 36 h post-reperfusion, which were significantly increased in the IRI group and reduced with DZNep treatment. Additionally, the reduction in SCr and BUN concentrations was especially significant in the IR+DZNep group (Figure 1A). The survival rate, regardless of the time of DZNep administration, improved after 7 days (Figure 1B). H&E staining also demonstrated that DZNep could alleviate kidney injury after IRI. The 4-point scoring system was also used to evaluate tissue injury and found that injury scores were lower in DZNep treated group than in the IR group (Figure 1C). These results indicated that DZNep could alleviate renal IRI, especially through continued use from before to after surgery. In addition, DZNep had long-term effects on improving the survival rate.

### DZNep Decreased Proinflammatory Responses and Inhibited Inflammatory Reactions in IRI

**Figure 2:**
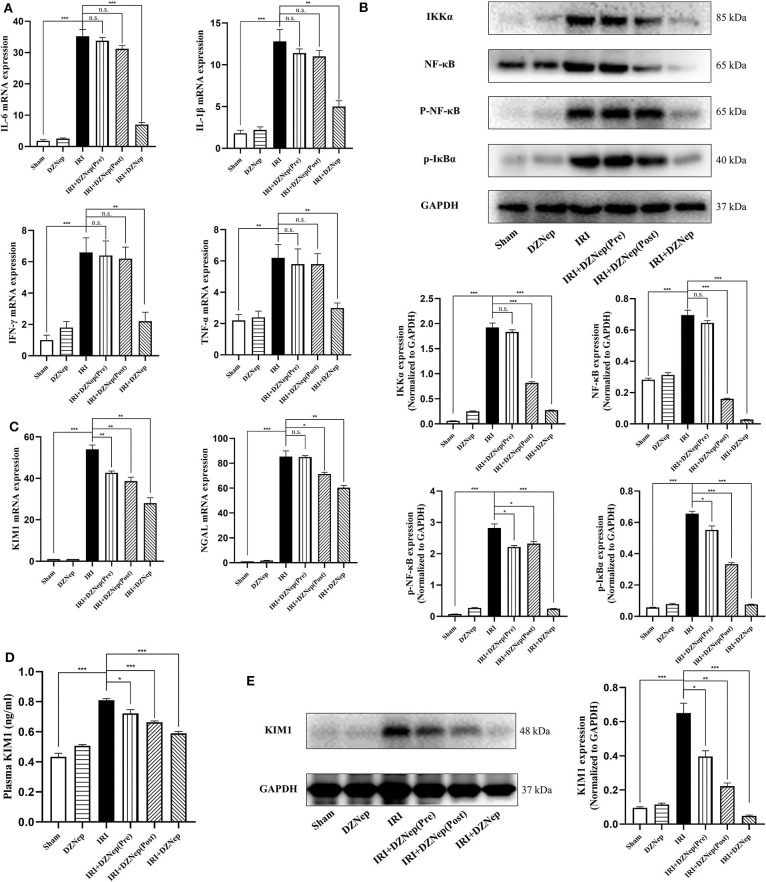
DZNep decreased the expression of inflammatory agents and injury relative molecules in renal IRI. **(A)** The relative mRNA expression level of IL-6, IL-10, IFN-γ, and TNF-α in mice kidney (*n* = 6). **(B)** Western blot analysis of protein expression in NF-κB signaling pathway (*n* = 6). The expression level was normalized to GAPDH. **(C)** Relative mRNA expression of KIM-1 and NGAL in mice kidneys (*n* = 6). **(D)** KIM-1 expression level in plasma (*n* = 6). **(E)** Western blot analysis of protein expression of KIM-1 in mice kidneys. The expression level was normalized to GAPDH. ^n.s.^*P* ≥ 0.05, *P < 0.05, **P < 0.01, ***P < 0.001.

The mRNA expression of proinflammatory cytokines IL-6, IL-1β, IFN-γ, and TNF-α increased after IRI and was reduced by DZNep treatment (Figure 2A). After IRI, the expression of the NF-κB signaling pathway was found to be activated. According to our results, IKKα, NF-κB, p-NF-κB, and p-IκBα differed significantly between the IR and sham groups, and decreased after DZNep treatment (Figure 2B). The realtime PCR, ELISA, and western blot analyses revealed a downregulation of KIM-1 in the kidney and plasma after treatment with DZNep, regardless of when treatment was initiated (Figures 2C–E). Another indicator for renal injury, neutrophil gelatinase-associated lipocalin (NGAL), was only downregulated in the post- and continued-treated DZNep groups (Figure 2C). These results confirmed an anti-inflammatory and mitigatory effect of DZNep in renal IRI. However, pretreatment with DZNep was insufficient to suppress inflammation. The continued-treated DZNep group presented the most significant protective effect.

### DZNep Reduced the Activation of CD4^+^ T Cells and Inhibited TIM-1 Expression

**Figure 3:**
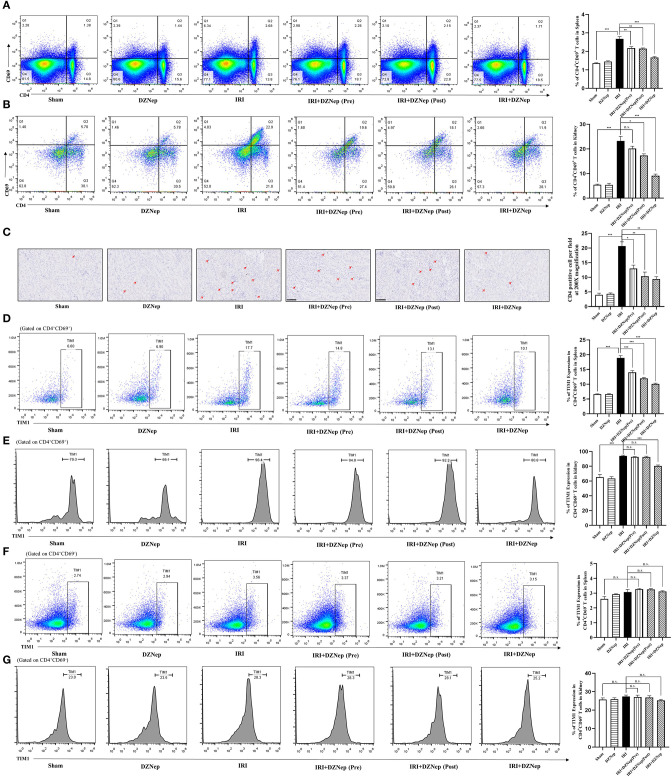
DZNep downregulated TIM-1 expression in activated CD4^+^T cells after IRI. **(A)** CD4^+^CD69^+^T cells in spleen were gated using flowcytometry. **(B)** The proportion of CD4^+^CD69^+^T cells in mice kidney. **(C)** Immunochemistry stained CD4^+^T cells in kidney. Histogram showed the average cell count of CD4^+^T cells per field (*n* = 18, 200X). **(D)** TIM-1 level in CD4^+^CD69^+^ T cells of mice spleen. **(E)** TIM-1 level in CD4^+^CD69^+^ T cells of mice kidney. **(F)** TIM-1 level in CD4^+^CD69^−^ T cells of mice spleen. **(G)** TIM-1 level in CD4^+^CD69^−^ T cells of mice kidney. ^n.s.^*P* ≥ 0.05, *P < 0.05, **P < 0.01, ***P < 0.001.

It has been reported that DZNep can induce apoptosis of activated T cells in a bone marrow transplantation model. Thus, we investigated the effect and relative mechanism of DZNep on CD4^+^ T cells in renal IRI. Flow cytometry showed that CD4^+^CD69^+^ T cells (activated T helper cells) were downregulated in the spleen and kidney after treatment with DZNep (Figures 3A,B), indicating a significant impairment in the activation of CD4^+^ T cells induced by DZNep. Immunohistochemistry also confirmed a significant decline in CD4^+^ cells in DZNep-treated IRI kidneys (Figure 3C).

Since TIM-1 is involved in the activation of CD4^+^ T cells, we evaluated the expression level of TIM-1 in CD4^+^CD69^+^ and CD4^+^CD69^−^ T cells (activated and inactivated T cells, respectively). As expected, TIM-1 was elevated in CD4^+^CD69^+^ T cells after IRI in both the spleen and kidney (Figures 3D,E), but remained unchanged in CD4^+^CD69^−^ T cells (Figures 3F,G). In addition, DZNep significantly downregulated TIM-1 expression in CD4^+^CD69^+^ T cells (Figures 3D,E) but had no effect on CD4^+^CD69^−^ T cells (Figures 3F,G). Thus, we could infer that TIM-1 plays a role in the activation of CD4+ T cells in IRI, and DZNep impaired the activation of CD4^+^ T cells by inhibiting the expression of TIM-1 on the surface. Interestingly, despite the trend, pretreatment of DZNep did not impair T cell activation and downregulate TIM-1 expression significantly in the kidney (Figures 3B,E), which was in accordance with SCr, BUN, and other injury related indicators.

### DZNep May Impair the Activation of T Cells *via* Direct and Indirect Pathways

**Figure 4:**
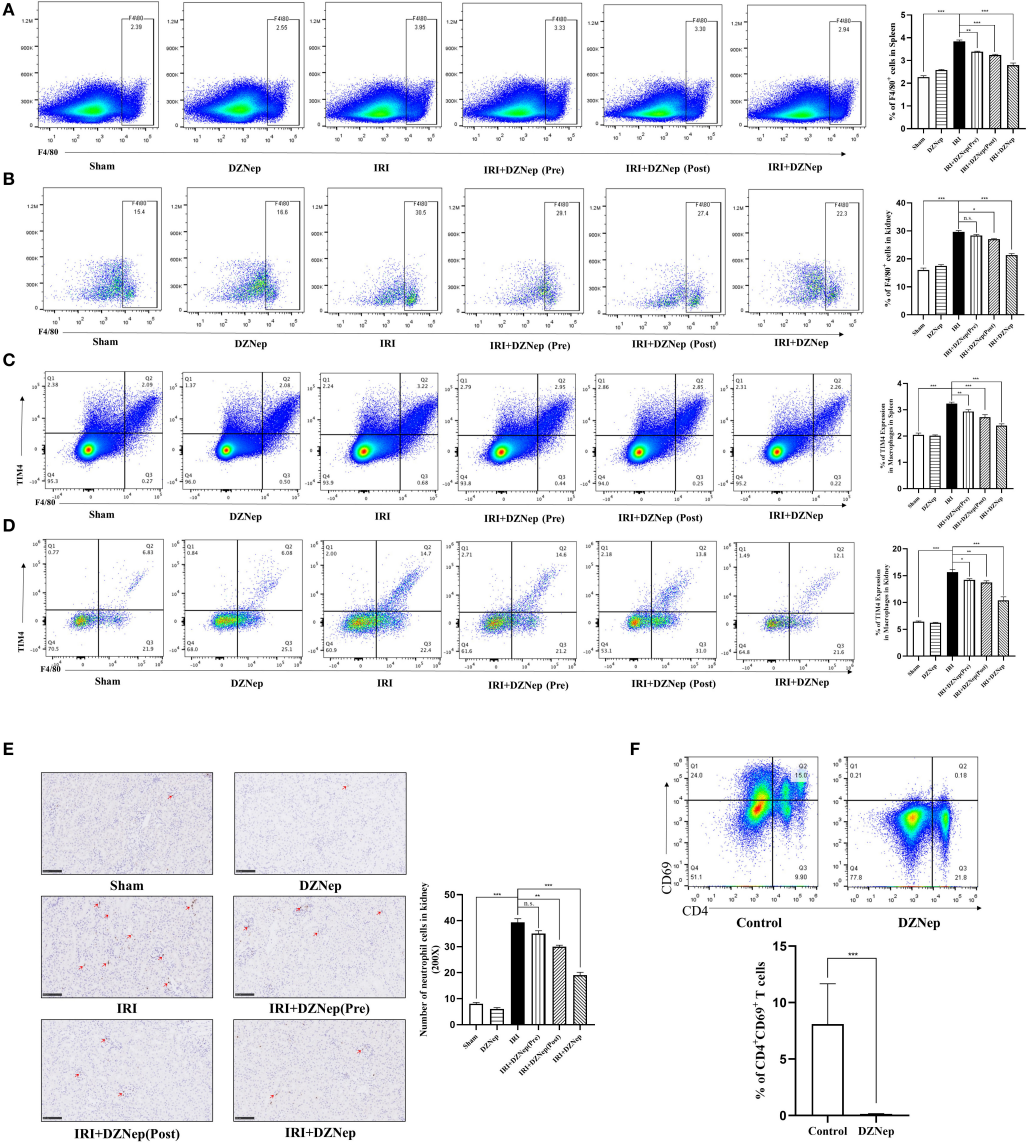
DZNep could impair activation of T cells by direct and indirect pathway. **(A)** The proportion of macrophages in mice spleen. **(B)** The proportion of macrophages in mice kidney. **(C)** TIM-4 level in macrophages of mice spleen. **(D)** TIM-4 level in macrophages of mice kidney. **(E)** MPO staining of neutrophils in mice kidney sections and neutrophil count in each group (*n* = 18, 200X). **(F)** Flow-cytometry evaluated the proportion of CD4^+^CD69^+^ T cells in spleen cells after 3 days culture with absence or presence of DZNep. ^n.s.^*P* ≥ 0.05, *P < 0.05, **P < 0.01, ***P < 0.001.

Since DZNep can widely affect other major inflammatory cells, such as macrophages and neutrophils, we conducted further experiments to determine whether DZNep impairs the activation of T cells via direct or indirect pathways. Macrophages accumulated in the IRI spleen and kidney, and this accumulation was mitigated after treatment with DZNep (Figures 4A,B). Notably, TIM-4, the ligand of TIM-1, was also significantly downregulated in macrophages (Figures 4C,D) after treatment with DZNep, indicating that DZNep may impair T cell activation by affecting the interaction of macrophages and T cells by modulating the TIM-1-TIM-4 axis. In addition, Neutrophils showed a similar trend in the kidney (Figure 4E). To determine whether DZNep has a direct effect on T cells, we cultured activated mouse splenic T cells (activated by conA) with or without DZNep. T cell activation was still impaired without interaction with other cells after treatment with DZNep (Figure 4F). Thus, DZNep has a direct effect on T cell activation.

### DZNep Downregulated the NF-κB Signaling Pathway in TCMK-1 Cells

**Figure 5:**
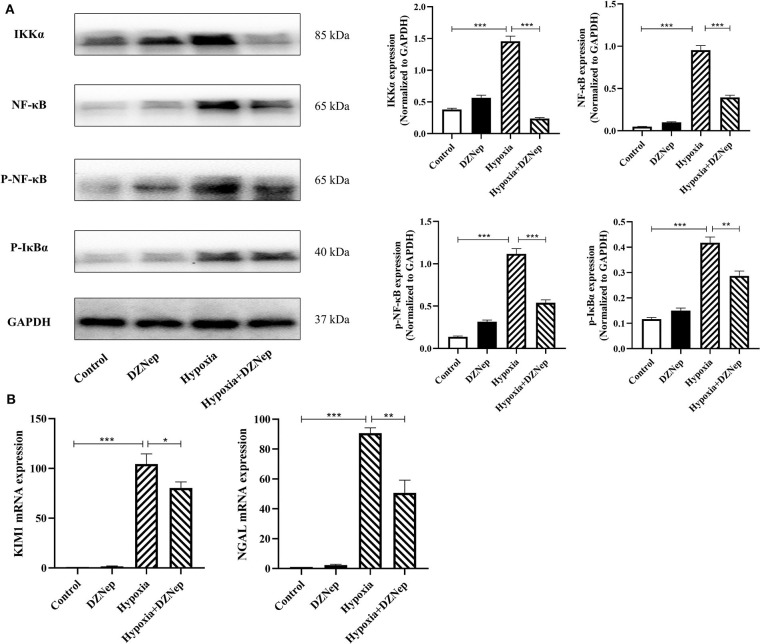
DZNep downregulated expression of KIM-1 and NF-κB signaling pathway in TCMK-1 cells. **(A)** Realtime PCR revealed KIM-1 and NGAL relative mRNA expression. **(B)** Western blot analysis of NF-κB signaling pathway protein expression in TCMK-1 cells. The expression level was normalized to GAPDH. *P < 0.05, **P < 0.01, ***P < 0.001.

TCMK-1 cells were used to confirm the anti-inflammatory effect of DZNep *in vitro*. TCMK-1 cells were exposed to H/R. KIM-1 and NGAL expression as well as components of the NF-κB signaling pathway were all downregulated in DZNep-treated TCMK-1 cells (Figures 5A,B). Therefore, DZNep can protect the renal tubular cells from exacerbated inflammation directly in renal IRI.

## Discussion

In this study, we investigated the therapeutic effects and optimal administration time of DZNep in renal IRI for the first time. DZNep treatment from before to after surgery showed adequate renal protective effects, while treating with DZNep only before surgery was insufficient to alleviate injury. After treatment with DZNep, the renal injury and inflammatory reaction were ameliorated and T cell activation was directly and significantly impaired by DZNep, with a downregulation of TIM-1 transcription in activated T cells and decreased TIM-4 expression in macrophages.

IRI is the common cause of acute renal failure in both allograft and native kidneys. Researchers have found that T cells play a key role in renal IRI (8). Depletion of T cells improved the outcomes of IRI and was believed to be a promising therapeutic target in preventing allograft rejection. However, complete depletion of T cells is unrealistic for clinical practice and experiments using knockout mice may not reflect the intervention effects in wild-type mice or humans. With advances in the understanding of chromatin remodeling and epigenetic changes during T cell development and function, epigenetic therapy targeting T cells is rapidly developing. Histone methylation is more stable than other histone modification forms, which could provide a long-term maintenance of chromatin states that cannot be silenced by DNA methylation (17, 21, 22). Therefore, medication targeting histone methylation in T cells may be more reliable for long-term intervention among post-transplant patients. EZH2 was associated with cytokine gene expression in T cells (13, 23). As an inhibitor of histone EZH2, DZNep was reported to have protective effects in ischemic brain injury (18) and tubulointerstitial fibrosis (24). In our study, DZNep ameliorated the renal IRI and injury of the renal tubular cells *in vivo* and *in vitro* and such protective effects persisted and improved the survival rate in our 3-days follow-up. In addition, the activation of T cells was still impaired when cultured solely with DZNep, indicating a direct action of DZNep on T cell activation, which has not been previously reported. Therefore, DZNep may become a promising therapeutic for modulating the function of T cells in renal IRI.

The TIM-1-TIM-4 axis was suggested as a novel intervention target for IRI. Direct inhibition or induced depression of TIM-1 and TIM-4 protected against liver and cerebral IRI (25–29). Blocking the TIM-1-TIM-4 pathway by anti-TIM monoclonal antibody also diminished inflammation and improve apoptosis and survival in renal IRI (11). The TIM-1-TIM-4 axis is involved in the activation and proliferation of all T cells, including Tregs (29–32), and impacts the infiltration and activation of macrophages and neutrophils as well (10). Eventually, we found that TIM-1 was elevated in activated T cells after renal IRI, and DZNep treatment significantly downregulated TIM-1 expression. In addition, since TIM-4 is highly restricted to antigen-presenting cells, we also evaluated the expression level of TIM-4 in macrophages and found that TIM-4 expression in macrophages was in parallel with TIM-1. Macrophages were also recruited less to the kidney after treatment with DZNep. Thus, TIM-1 and TIM-4 may be involved in the interaction of macrophages and T cells, and may be an indirect mechanism for DZNep-mediated impairment of T cell activation.

Another aspect of our study was to determine the optimal administration time of DZNep. To our surprise, pretreatment with DZNep showed an unsatisfactory protective effect against renal injury, while post-treatment with DZNep significantly inhibited the injury. Based on this phenomenon, we suspect that the renal protective effect of DZNep mainly involves blocking the deterioration process after the occurrence of injury, rather than prompting cells to undergo adaptive changes in advance. Combining the fact that TIM-1 expression was only downregulated in activated T cells while it remained unchanged in naïve T cells, we inferred that DZNep had no effect on T cells in the resting state and can block the activation process of T cells by inhibiting TIM-1 expression. However, the underlying mechanism requires further exploration.

**Figure 6:**
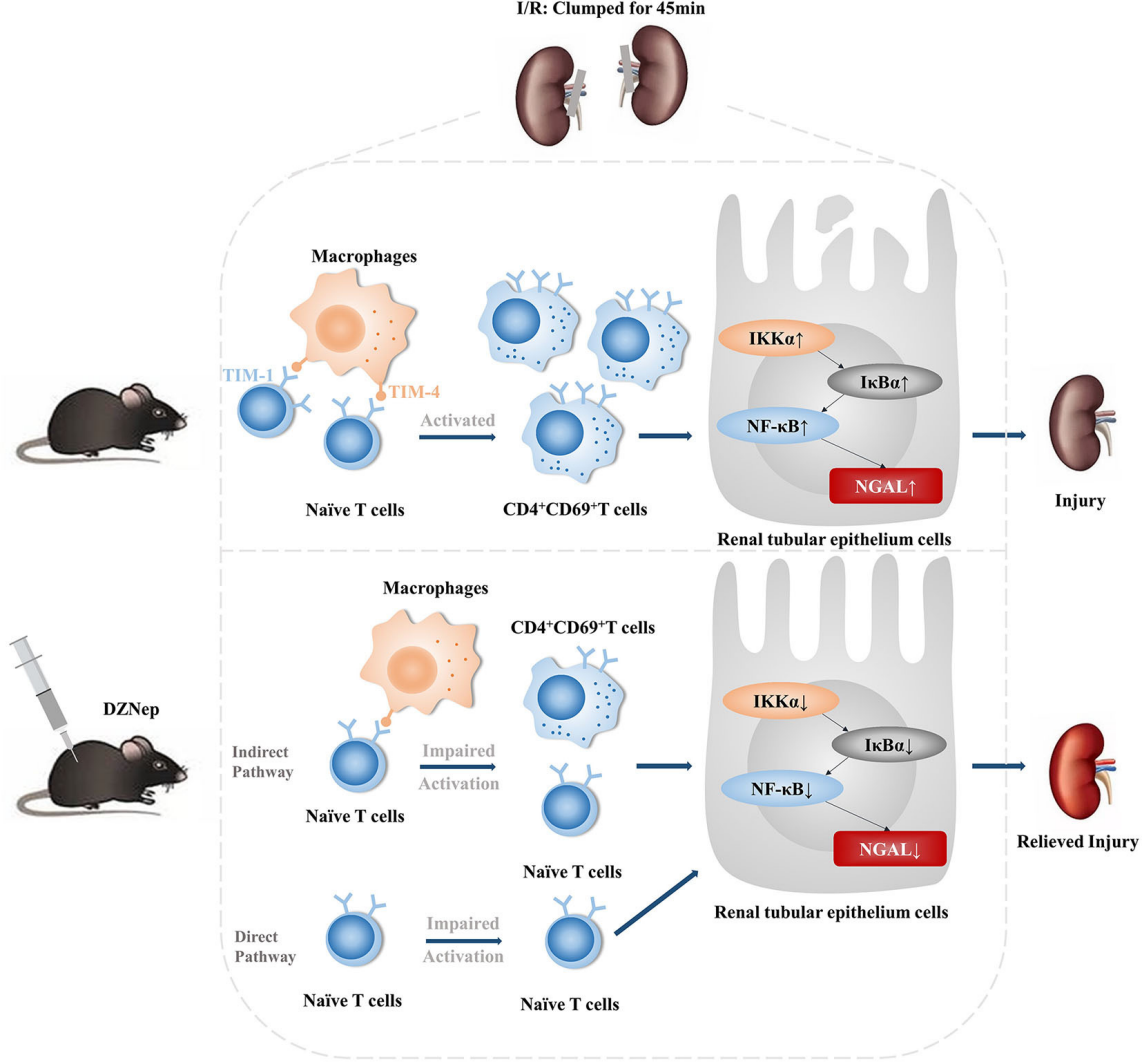
The pictorial summary of DZNep on renal IRI. DZNep treatment in I/R mice could significantly downregulated TIM-1 expression on naïve T cells and impaired their activation. DZNep could alleviate mice renal IRI by inhibiting T cell activation in direct and indirect pathway. The indirect pathway might be involved in the impaired interaction between T cells and macrophages by TIM-1: TIM-4 axis. And DZNep could have a better protective effect when used postoperatively.

In conclusion, our study demonstrated that DZNep ameliorated acute renal injury and inflammatory responses in a renal IRI murine model by modulating CD4^+^ T cell activation. DZNep also inhibited the recruitment of neutrophils by CD4^+^ T cells. The modulation effect of DZNep on T cells may involve histone methylation and subsequent downregulation of TIM-1 (Figure 6).

## Data Availability Statement

All datasets generated for this study are included in the article/supplementary material.

## Ethics Statement

The animal study was reviewed and approved by Animal Ethical Committee of Zhongshan Hospital, Fudan University.

## Author Contributions

JL, LL, YZ, and RR: conceived the project, designed the project, extracted and analyzed data, and approved the final manuscript. YQ: drafted the manuscript. JiyW, YC, YJ, JinW, and RS: conducted the experiments. All authors contributed to the article and approved the submitted version.

## Conflict of Interest

The authors declare that the research was conducted in the absence of any commercial or financial relationships that could be construed as a potential conflict of interest.

## References

[1] Cravedi P Heeger PS. Corrigendum. Complement as a multifaceted modulator of kidney transplant injury. J Clin Invest. (2015) 125:1365. 10.1172/JCI81182 25729858 PMC4362224

[2] Ponticelli C. Ischaemia-reperfusion injury: a major protagonist in kidney transplantation. Nephrol Dial Transplant. (2014) 29:1134–40. 10.1093/ndt/gft488 24335382

[3] Schroppel B Legendre C. Delayed kidney graft function: from mechanism to translation. Kidney Int. (2014) 86:251–8. 10.1038/ki.2014.18 24522494

[4] Rabb H Daniels F O'Donnell M Haq M Saba SR Keane W. Pathophysiological role of T lymphocytes in renal ischemia-reperfusion injury in mice. Am J Physiol Renal Physiol. (2000) 279:F525–31. 10.1152/ajprenal.2000.279.3.F525 10966932

[5] Ysebaert DK De Greef KE De Beuf A Van Rompay AR Vercauteren S Persy VP. T cells as mediators in renal ischemia/reperfusion injury. Kidney Int. (2004) 66:491–6. 10.1111/j.1523-1755.2004.761_4.x 15253695

[6] Pinheiro HS Camara NO Noronha IL Maugeri IL Franco MF Medina JO. Contribution of CD4+ T cells to the early mechanisms of ischemia- reperfusion injury in a mouse model of acute renal failure. Braz J Med Biol Res. (2007) 40:557–68. 10.1590/S0100-879X2007000400015 17401500

[7] Xue C Liu Y Li C Li Y Yang T Xie L. Powerful protection against renal ischemia reperfusion injury by T cell-specific NF-κB inhibition. Transplantation. (2014) 97:391–6. 10.1097/01.TP.0000438622.89310.95 24398854

[8] Yokota N Daniels F Crosson J Rabb H. Protective effect of T cell depletion in murine renal ischemia-reperfusion injury. Transplantation. (2002) 74:759–63. 10.1097/00007890-200209270-00005 12364852

[9] Lee J Phong B Egloff AM Kane LP. TIM polymorphisms–genetics and function. Genes Immun. (2011) 12:595–604. 10.1038/gene.2011.75 22048452 PMC3281969

[10] Zhang Y Ji H Shen X Cai J Gao F Koenig KM. Targeting TIM-1 on CD4 T cells depresses macrophage activation and overcomes ischemia-reperfusion injury in mouse orthotopic liver transplantation. Am J Transplant. (2013) 13:56–66. 10.1111/j.1600-6143.2012.04316.x 23137033 PMC3535503

[11] Rong S Park JK Kirsch T Yagita H Akiba H Boenisch O. The TIM-1:TIM-4 pathway enhances renal ischemia-reperfusion injury. J Am Soc Nephrol. (2011) 22:484–95. 10.1681/ASN.2010030321 21355054 PMC3060442

[12] Mas VR Le TH Maluf DG. Epigenetics in kidney transplantation: current evidence, predictions, and future research directions. Transplantation. (2016) 100:23–38. 10.1097/TP.0000000000000878 26356174 PMC6843994

[13] Koyanagi M Baguet A Martens J Margueron R Jenuwein T Bix M. EZH2 and histone 3 trimethyl lysine 27 associated with Il4 and Il13 gene silencing in Th1 cells. J Biol Chem. (2005) 280:31470–7. 10.1074/jbc.M504766200 16009709

[14] Tumes DJ Onodera A Suzuki A Shinoda K Endo Y Iwamura C. The polycomb protein Ezh2 regulates differentiation and plasticity of CD4^+^ T helper type 1 and type 2 cells. Immunity. (2013) 39:819–32. 10.1016/j.immuni.2013.09.012 24238339

[15] Zhou J Bi C Cheong LL Mahara S Liu SC Tay KG. The histone methyltransferase inhibitor, DZNep, up-regulates TXNIP, increases ROS production, and targets leukemia cells in AML. Blood. (2011) 118:2830–39. 10.1182/blood-2010-07-294827 21734239

[16] Jiang X Lim CZ Li Z Lee PL Yatim SM Guan P. Functional characterization of D9, a novel deazaneplanocin A (DZNep) analog, in targeting acute myeloid leukemia (AML). PLoS ONE. (2015) 10:e0122983. 10.1371/journal.pone.0122983 25928216 PMC4415792

[17] Miranda TB Cortez CC Yoo CB Liang G Abe M Kelly TK. DZNep is a global histone methylation inhibitor that reactivates developmental genes not silenced by DNA methylation. Mol Cancer Ther. (2009) 8:1579–88. 10.1158/1535-7163.MCT-09-0013 19509260 PMC3186068

[18] Chen J Zhang M Zhang X Fan L Liu P Yu L. EZH2 inhibitor DZNep modulates microglial activation and protects against ischaemic brain injury after experimental stroke. Eur J Pharmacol. (2019) 857:172452. 10.1016/j.ejphar.2019.172452 31202798

[19] He S Wang J Kato K Xie F Varambally S Mineishi S. Inhibition of histone methylation arrests ongoing graft-versus-host disease in mice by selectively inducing apoptosis of alloreactive effector T cells. Blood. (2012) 119:1274–82. 10.1182/blood-2011-06-364422 22117046 PMC3338164

[20] Hesketh EE Czopek A Clay M Borthwick G Ferenbach D Kluth D. Renal ischaemia reperfusion injury: a mouse model of injury and regeneration. J Vis Exp. (2014) 51816. 10.3791/51816 24961244 PMC4188040

[21] Berger SL. The complex language of chromatin regulation during transcription. Nature. (2007) 447:407–12. 10.1038/nature05915 17522673

[22] Jenuwein T Allis CD. Translating the histone code. Science. (2001) 293:1074–80. 10.1126/science.1063127 11498575

[23] Lee CG Sahoo A Im SH. Epigenetic regulation of cytokine gene expression in T lymphocytes. Yonsei Med J. (2009). 50:322–30. 10.3349/ymj.2009.50.3.322 19568591 PMC2703752

[24] Mimura I Hirakawa Y Kanki Y Nakaki R Suzuki Y Tanaka T. Genome-wide analysis revealed that DZNep reduces tubulointerstitial fibrosis via down-regulation of pro-fibrotic genes. Sci Rep. (2018) 8:3779. 10.1038/s41598-018-22180-5 29491489 PMC5830881

[25] Zheng L Huang Y Wang X Wang X Chen W Cheng W. Inhibition of TIM-4 protects against cerebral ischaemia-reperfusion injury. J Cell Mol Med. (2019) 24:1276–85. 10.1111/jcmm.14754 31774937 PMC6991695

[26] Zhang Y Liu Y Chen H Zheng X Xie S Chen W. TIM-1 attenuates the protection of ischemic preconditioning for ischemia reperfusion injury in liver transplantation. Am J Transl Res. (2017) 9:3665–75. 28861157 PMC5575180

[27] Zhang Y Shen Q Liu Y Chen H Zheng X Xie S. Hepatic ischemic preconditioning alleviates ischemia-reperfusion injury by decreasing TIM4 expression. Int J Biol Sci. (2018) 14:1186–95. 10.7150/ijbs.24898 30123068 PMC6097479

[28] Zheng Y Wang L Chen M Liu L Pei A Zhang R Inhibition of T cell immunoglobulin and mucin-1 (TIM-1) protects against cerebral ischemia-reperfusion injury. Cell Commun Signal. (2019) 17:103 10.1186/s12964-019-0417-4 31438964 PMC6704646

[29] Ji H Liu Y Zhang Y Shen XD Gao F Busuttil RW. T-cell immunoglobulin and mucin domain 4 (TIM-4) signaling in innate immune-mediated liver ischemia-reperfusion injury. Hepatology. (2014) 60:2052–64. 10.1002/hep.27334 25066922 PMC4396987

[30] Mariat C Sanchez-Fueyo A Alexopoulos SP Kenny J Strom TB Zheng XX. Regulation of T cell dependent immune responses by TIM family members. Philos Trans R Soc Lond B Biol Sci. (2005) 360:1681–5. 10.1098/rstb.2005.1706 16147532 PMC1569540

[31] Meyers JH Chakravarti S Schlesinger D Illes Z Waldner H Umetsu SE. TIM-4 is the ligand for TIM-1, and the TIM-1-TIM-4 interaction regulates T cell proliferation. Nat Immunol. (2005) 6:455–64. 10.1038/ni1185 15793576

[32] Tan X Jie Y Zhang Y Qin Y Xu Q Pan Z. Tim-1 blockade with RMT1-10 increases T regulatory cells and prolongs the survival of high-risk corneal allografts in mice. Exp Eye Res. (2014) 122:86–93. 10.1016/j.exer.2014.02.019 24613782

